# Two-metal-edge extended X-ray absorption fine structure analysis of oxygen octahedral rotation in SrTiO_3_ using the reverse Monte Carlo method

**DOI:** 10.1107/S1600577525004515

**Published:** 2025-06-18

**Authors:** Kai Kamijo, Nobuo Nakajima, Dongxiao Fan, Andris Anspoks

**Affiliations:** a Graduate School of Advanced Science and Engineering Hiroshima University 1-3-1 Kagamiyama Hiroshima Higashi-Hiroshima739-8526 Japan; b Institute of Materials Structure Science High Energy Accelerator Research Organization (KEK) 1-1 Oho Tsukuba Ibaraki305-0801 Japan; c Institute of Solid State Physics University of Latvia Kengaraga Street 8 RigaLV-1063 Latvia; ESRF – The European Synchrotron, France

**Keywords:** two-metal-edge EXAFS, reverse Monte Carlo, SrTiO_3_, cubic-to-tetragonal phase transitions

## Abstract

A three-dimensional analysis method for X-ray absorption spectra, combining two-metal-edge extended X-ray absorption fine structure (EXAFS) analysis with reverse Monte Carlo simulations, was applied to study the cubic-to-tetragonal phase transition in SrTiO_3_ as a dielectric standard. This method allows for the evaluation of both the static distribution and the dynamic motion of atoms, including oxygen. The optimized clusters reveal that the TiO_6_ octahedron can rotate up to 2.7° below the phase transition temperature, driven by reduced *A*-site Sr dynamics. Within the framework of conventional EXAFS analysis, the high-precision determination of atomic positions using the two-metal-edge analysis for ternary materials enhances the detection of light elements, and the method is applicable to bimetallic oxides and nitrides.

## Abstract 2

Improved accuracy of the octahedral local rotation in SrTiO_3_ has been achieved by simultaneous two-metal-edge EXAFS refinement.

## 1. Introduction

Extended X-ray absorption fine structure (EXAFS) analysis is a powerful technique for extracting element-selective local structure information (Sayers *et al.*, 1971 ▸; Ravel & Stern, 1995 ▸; Rehr & Albers, 2000 ▸) and is widely used in material characterization research owing to its versatile experimental setup (Mizumaki *et al.*, 2009 ▸; Nagasaka *et al.*, 2014 ▸; Kato *et al.*, 2021 ▸). However, despite its strengths, resolving the three-dimensional structure of materials remains a challenging task, particularly for those involving light elements, owing to complexities introduced by their larger thermal vibration and by photoelectron multiple scattering (MS) (Filipponi *et al.*, 1995 ▸). To address these challenges, we revisit a three-dimensional analysis method for EXAFS spectra using reverse Monte Carlo (RMC) simulations which has been intensively studied (Mcgreevy & Pusztai, 1988 ▸; Gurman & McGreevy, 1990 ▸; Winterer, 2000 ▸; Di Cicco & Trapananti, 2005 ▸). This approach involves the simultaneous fitting of two or more spectra, which effectively addresses the difficulties associated with light-element analysis and MS. Prior to the incorporation of the RMC method, the multiple-edge analysis has already been developed in a sophisticated approach by Di Cicco and coworkers with the *GNXAS* code, starting from multiple core-edge spectra analysis for Sn *L*_1_-, *L*_2_- and *L*_3_-edges (Di Cicco, 1996 ▸), followed by complex metalloproteins containing Fe and Cu (Zhang *et al.*, 1996 ▸), and amorphous molten salts of CuBr and RbBr (Di Cicco *et al.*, 1997 ▸). The multiple-edge RMC EXAFS refinement is also examined by the same group ranging from solid to liquid phases (Di Cicco & Trapananti, 2005 ▸; Cicco *et al.*, 2018 ▸; Di Cicco & Iesari, 2022 ▸).

In the present study, we applied this approach to a ternary bimetallic oxide using the *EvAX* code developed by Timoshenko *et al.* (2012 ▸), which utilizes hundreds of scattering paths without requiring special assumptions, thus enabling the comprehensive consideration of MS contributions. The use of the Morlet wavelet transform (WT), first introduced into EXAFS analysis by Funke *et al.* (2007 ▸), is yet another advantage of this code. The EXAFS signals together with scattering paths are obtained using the *FEFF8.5L* code without cluster size constraints (Rehr & Albers, 2000 ▸).

Given the ongoing debate about local versus long-range structural order in perovskites, strontium titanate (SrTiO_3_; STO), a prototypical dielectric material, was selected as the first model compound to evaluate the advantages of the two-metal-edge analysis using the RMC approach. At room temperature, STO adopts a cubic (*Pm*3*m*) perovskite-type structure. On cooling below *T*_C_ = 105 K, it undergoes a second-order phase transition to a tetragonal symmetry (*I*4/*mcm*) characterized by octahedral rotation, driven by the condensation of the R_25_ phonon mode at the Brillouin zone boundary (Fleury *et al.*, 1968 ▸; Shirane & Yamada, 1969 ▸; Kiat & Roisnel, 1996 ▸; Hayward & Salje, 1999 ▸). Neutron diffraction (Hui *et al.*, 2005 ▸) and electron paramagnetic resonance spectroscopy (Hayward & Salje, 1999 ▸) analyses report that the rotation angle reaches approximately 2°. This minor rotation, which is not captured by single-edge RMC analysis at either the Ti *K*- or the Sr *K*-edge (Anspoks *et al.*, 2015 ▸; Anspoks *et al.*, 2020 ▸), serves as a benchmark for the performance testing of two-metal-edge analysis methods. Furthermore, the bonding interactions between oxygen and *A*-site alkaline earth metals in *A*TiO_3_ perovskites play a key role in the thermal stability of spontaneous polarization, which is reflected in differences in Curie temperature (Cohen, 1992 ▸; Suasmoro *et al.*, 2000 ▸; Tkacz-Śmiech *et al.*, 2003 ▸; Schiemer *et al.*, 2016 ▸). However, obtaining EXAFS data for the oxygen *K*-edge is difficult owing to its overlap with the Ti *L*_1_-edge. Inelastic scattering processes, such as non-resonant inelastic X-ray scattering and electron energy-loss spectroscopy, would provide complementary information on the local environment of oxygen, although these techniques tend to require complex apparatus or an elaborate sample preparation. In this study, temperature-dependent EXAFS spectra were recorded at the Sr-*K* and Ti-*K* absorption edges. Simultaneous fitting of both spectra was then performed over a wide temperature range across *T*_C_ using the RMC method to elucidate the local oxygen structure in STO. The methodology has potential applications in studying light-element dynamics in bimetallic oxides and nitrides.

## 2. Experimental and data analysis

SrTiO_3_ powders (4N, Kojundo Chemical, Japan) were mixed with boron nitride binder (99.5%, Ishizu Pharmaceutical, Japan) to prepare pellets. The EXAFS spectra at the Sr-*K* and Ti-*K* edges of STO were measured in transmission mode over the temperature range 26–300 K on beamline BL12C at the Photon Factory, High Energy Research Organization (KEK-PF). Synchrotron radiation from a bending magnet was monochromated using an Si(111) double-crystal monochromator. The intensities of incident and transmitted X-rays were recorded using ionization chambers. Background subtraction, normalization and Fourier transformation (FT) were performed using the *ATHENA* program (Ravel & Newville, 2005 ▸).

The EXAFS spectra extracted from the program were analyzed using the RMC method embedded in the *EvAX* code (Timoshenko *et al.*, 2012 ▸), which optimizes a three-dimensional atomic cluster model based on multiple EXAFS spectra through iterative processes. This approach enables stereoscopic analyses, including measurements of bond angles among three atoms and local distortions, with improved accuracy compared with single-edge EXAFS analysis. In this study, a supercell comprising 6 × 6 × 6 tetragonal unit cells (4320 atoms) with periodic boundary conditions was used for all temperature data. The lattice constants and initial atomic positions for the supercell were obtained from the literature (Hui *et al.*, 2005 ▸) and interpolated linearly. To investigate the structural changes without assumptions, even in the cubic phase above *T*_C_ = 105 K, a tetragonal unit cell was assumed, with *c*/*a* = 1 and ϕ = 0, where ϕ represents the rotation angle of the TiO_6_ octahedron along the *c* axis. The number of scattering paths used in the calculations was 200 for the Sr-*K* edge and 434 for the Ti-*K* edge, including MS effects up to the sixth order. Less significant paths, contributing less than 0.4% and 0.2% to the Sr-*K* and Ti-*K* edges, respectively, were omitted to save computation time and memory space. The atomic motion in each RMC step was 0.001 Å and the total maximum displacement was limited to less than 0.4 Å to avoid unphysical features. Simulated spectra were calculated at every step. More than 10000 RMC steps were satisfactory to reach equilibrium. In this study, the total number of the RMC steps was set to 18500 to confirm convergence. Three or four RMC runs were performed with different random number seeds. Prior to the simulations for all temperatures, seven to eight test simulations were conducted for both Sr and Ti edges independently using the lowest 26 K spectra to identify energy thresholds (*E*_0_) that minimize residuals between experimental and simulated EXAFS spectra. The Morlet WT was applied to compare the experimental and theoretical EXAFS spectra in *k*/*R*-space ranges extending from 3.0–15.6 Å^−1^/1.2–5.8 Å and 3.5–16.5 Å^−1^/1.0–6.0 Å for the Sr-*K* and Ti-*K* edges, respectively (Timoshenko & Kuzmin, 2009 ▸). Here the use of the RMC method enables the incorporation of broader fitting ranges compared with conventional EXAFS analysis.

## 3. Results and discussion

### 3.1. EXAFS spectra

**Figure 1:**
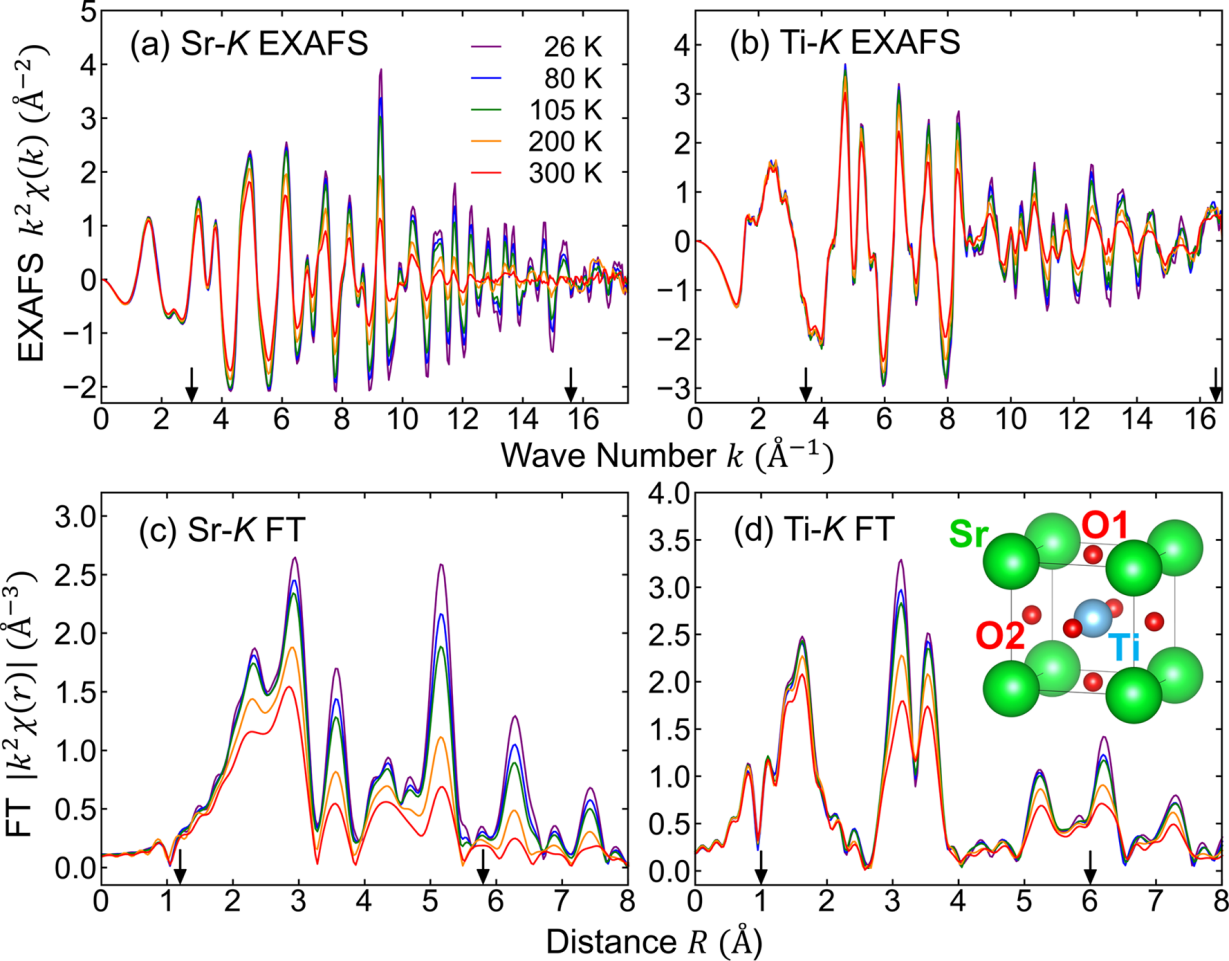
*k*^2^-weighted EXAFS spectra recorded at the (*a*) Sr-*K* and (*b*) Ti-*K* edges at five selected temperatures (26–300 K) and (*c*)–(*d*) their corresponding FT spectra. Arrows indicate the fitting ranges for *k*-space and *R*-space. The inset in (*d*) depicts the crystal structure of SrTiO_3_.

The Sr-*K* and Ti-*K* edge *k*^2^-weighted EXAFS spectra collected and their corresponding FTs at various temperatures are displayed in Figs. 1 ▸(*a*) and 1 ▸(*c*), and Figs. 1 ▸(*b*) and 1 ▸(*d*), respectively, where *k* denotes the photoelectron wavenumber. The arrows in Figs. 1 ▸(*a*)–1 ▸(*d*) indicate the fitting ranges for the *k*-space and *R*-space used in the RMC simulations. The low-noise EXAFS spectra facilitate high-resolution analysis (Stern, 2001 ▸). Notably, the temperature dependence of the Sr—O and Sr—Sr peaks in the Sr-*K* FT spectra is more pronounced than that of the Ti—O and Ti—Ti peaks in the Ti-*K* FT spectra, likely owing to the significantly greater strength of the Ti—O bond compared with that of the ionic Sr—O bond. The signals corresponding to the first (Sr—O: 2.0–2.5 Å) and second (Sr—Ti: 2.5–3.2 Å) coordination spheres of Sr overlap and cannot be resolved, as depicted in Fig. 1 ▸(*c*). Similarly, the second (Ti—Sr: 2.7–3.3 Å) and third (Ti—Ti: 3.3–3.8 Å) coordination spheres in the Ti-*K* FT spectra also overlap, as illustrated in Fig. 1 ▸(*d*). The contribution of MS effects, such as the linear Ti—O—Ti path, becomes significant beyond 3 Å. While traditional fitting procedures can account for MS paths, incorporating the interdependencies of the fitting parameters becomes increasingly complex. Furthermore, treating all parameters for all paths as free parameters can lead to unrealistic results.

### 3.2. Wavelet transform

**Figure 2:**
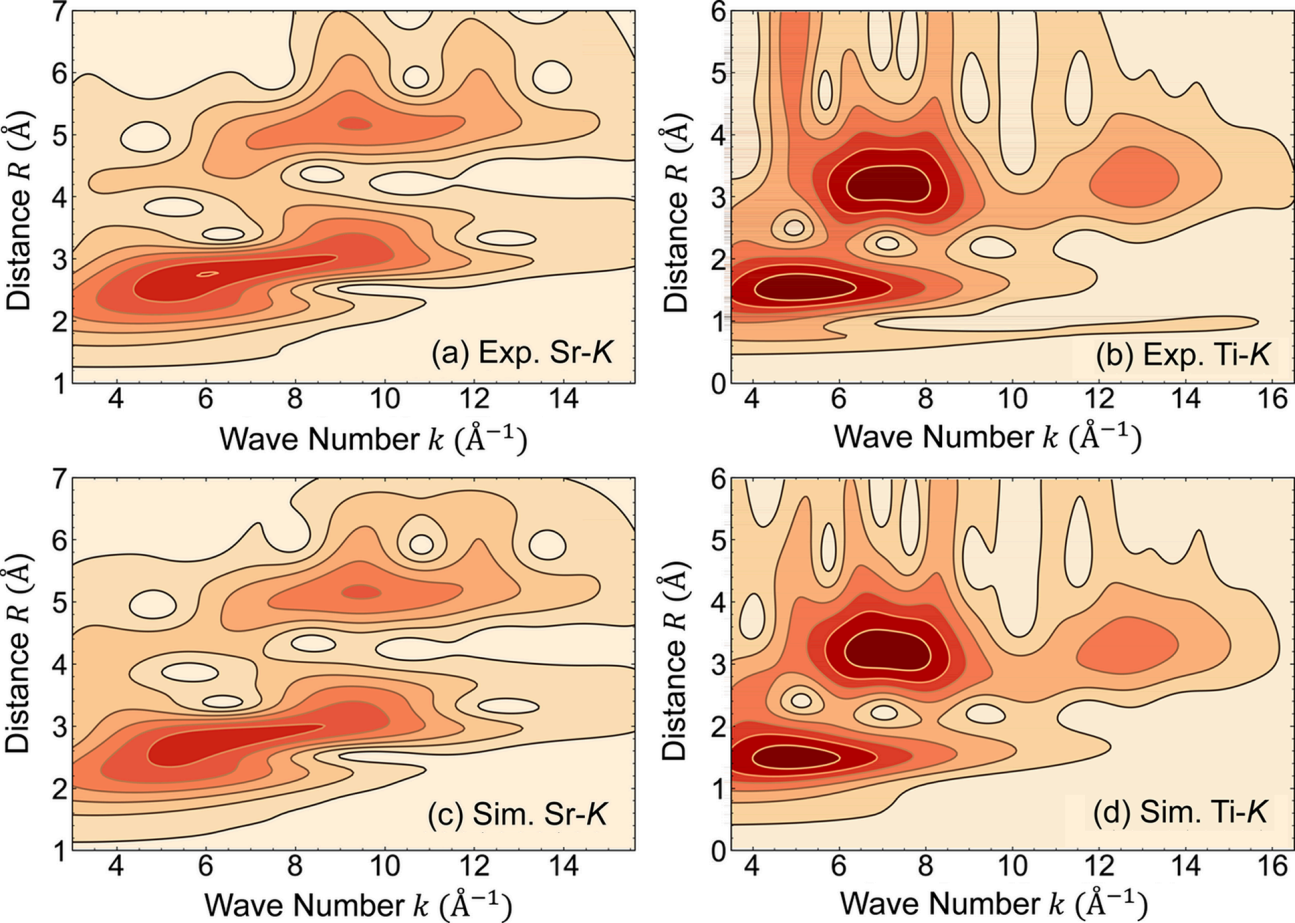
WTs of (*a*) Sr-*K* and (*b*) Ti-*K* edge EXAFS spectra at 26 K, and (*c*) and (*d*) WTs obtained from RMC-derived clusters.

The Morlet WT was used to compare experimental and theoretical EXAFS spectra over the following ranges: 3.0–15.6 Å^−1^ (Sr-*K*) and 3.5–16.5 Å^−1^ (Ti-*K*) in *k*-space and 1.2–5.8 Å (Sr-*K*) and 1.0–6.0 Å (Ti-*K*) in *R*-space, as depicted in Fig. 2 ▸. The upper two panels present experimental patterns, and the bottom two panels show simulated patterns. The WT generates a two-dimensional representation of periodic signals localized in *k*- and *R*-spaces, facilitating the identification of MS effects and reducing noise (Timoshenko & Kuzmin, 2009 ▸). For STO, the signals corresponding to Ti—Ti single scattering and Ti—O—Ti double scattering at the Ti-*K* edge are well separated at a distance of approximately 3.2 Å. The simulated WTs derived from the RMC clusters are in good agreement with the experimental patterns within the fitting range for both edges.

### 3.3. Radial distribution functions

**Figure 3:**
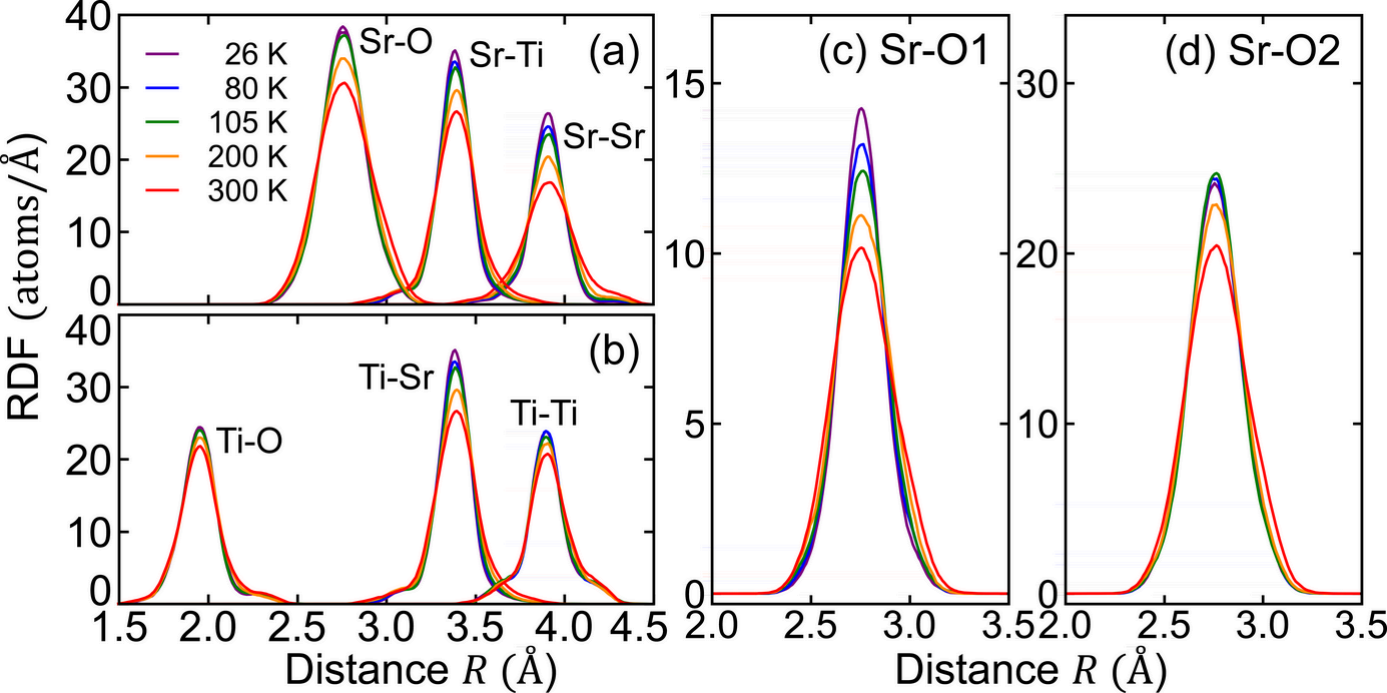
RDFs around (*a*) Sr and (*b*) Ti at five selected temperatures obtained from the RMC clusters. Oxygen-site-resolved RDFs for (*c*) Sr—O1 and (*d*) Sr—O2 are also shown. The cluster includes 2592 Sr—Sr, 2592 Ti—Ti, 6912 Sr—Ti, 6912 Sr—O1, 3456 Sr—O2, 6912 Ti—O1 and 1728 Ti—O2 atomic pairs.

Temperature-dependent radial distribution functions (RDFs) around Sr and Ti, obtained from the RMC-refined clusters, are presented in Figs. 3 ▸(*a*) and 3 ▸(*b*), respectively. The Sr—Ti and Ti—Sr distributions are identical, a direct result of using the same clusters at each temperature. The Ti-centered RDFs (Ti—O and Ti—Ti) are non-symmetric, in contrast to the symmetric Sr-centered RDFs (Sr—O and Sr—Sr). Moreover, the Ti-centered RDFs demonstrate weaker temperature dependence compared with the Sr-centered RDFs, consistent with the trends observed in the raw EXAFS data in Fig. 1 ▸. The comparison between Sr—O1 and Sr—O2 RDFs offers intriguing insights, where O1 and O2 represent the apical oxygen in the SrO plane and the planar oxygen in the TiO_2_ plane, respectively [refer to the inset of Fig. 1 ▸(*d*)]. As depicted in Figs. 3 ▸(*c*) and 3 ▸(*d*), the peak height of the Sr—O1 RDF increases gradually with decreasing temperature, while that of the Sr—O2 RDF remains unchanged below *T*_C_ = 105 K.

### 3.4. Mean square relative displacements

**Figure 4:**
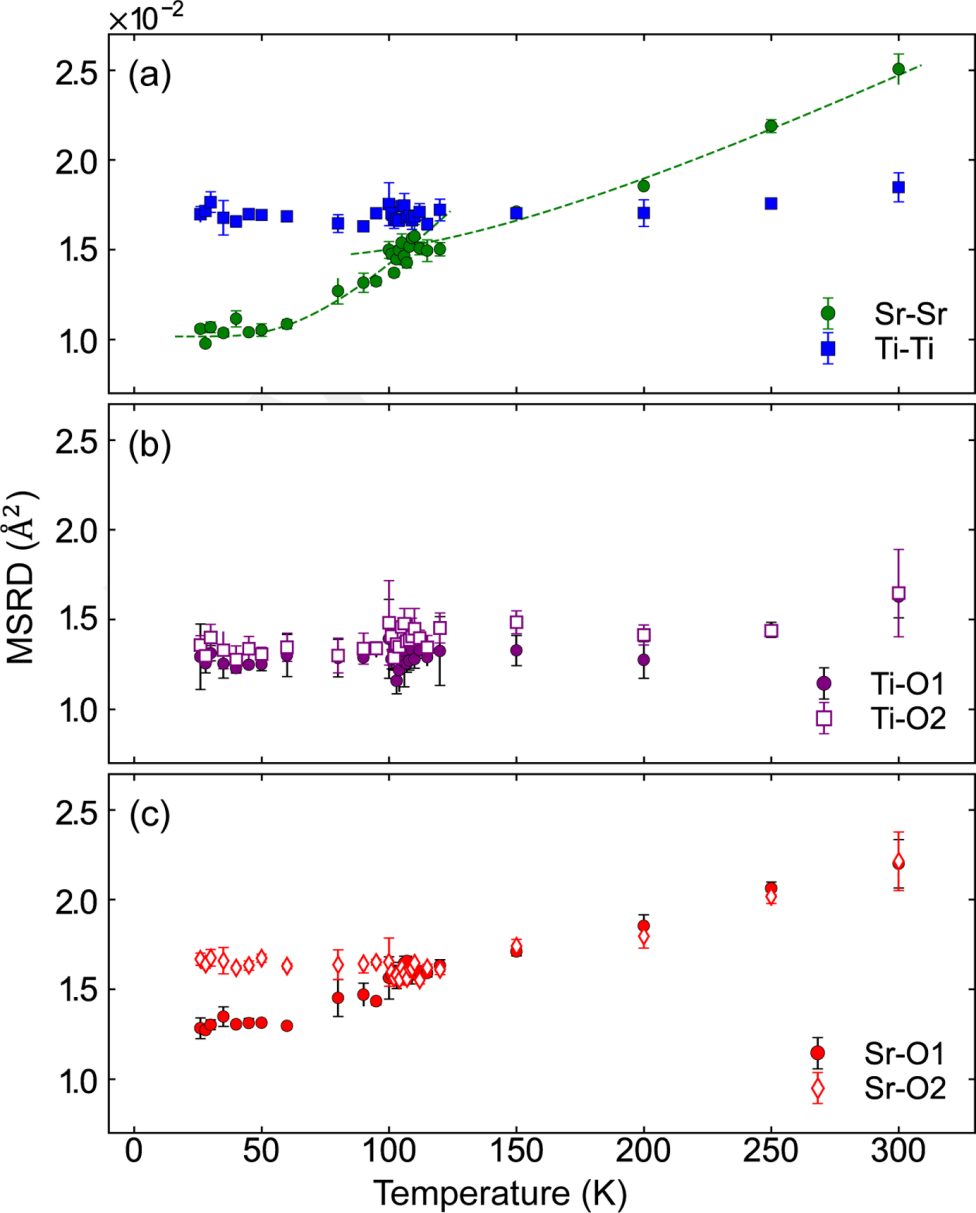
Temperature dependence of the MSRD for various atomic pairs: (*a*) Ti—Ti (blue squares) and Sr—Sr (green circles), (*b*) Ti—O1 (magenta filled circles) and Ti—O2 (magenta open squares), and (*c*) Sr—O1 (red filled circles) and Sr—O2 (red open diamonds). The dashed curves for Sr—Sr in (*a*) are provided as visual guides.

Fig. 4 ▸ presents the temperature dependence of the mean square relative displacements (MSRDs, also referred to as the Debye–Waller factor: σ^2^) for pairs of neighboring atoms at distance *r*. The MSRDs were calculated as the second moment of the RDFs using the following expression: σ^2^ = 〈(*r* − 〈*r*〉)^2^〉, where 〈…〉 denotes the average value (Fornasini & Grisenti, 2015 ▸). The error bars, including those in subsequent figures, were obtained from variation across three or four RMC runs. The MSRDs of Ti—Ti and Ti—O exhibit minimal temperature dependence below 250 K, consistent with the behavior of their corresponding RDFs. Notably, the MSRD of Sr—Sr decreases below *T*_C_, indicating a change in the vibrational mode associated with the cubic-to-tetragonal phase transition. Additionally, the MSRDs of Sr—O1 and Sr—O2 deviate below *T*_C_, in contrast to their identical slopes above *T*_C_. In general, σ^2^ is expressed as the sum of thermal and static factors: σ^2^ = 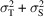. Given that the thermal factors for the same atomic pairs are expected to be identical, the difference between Sr—O1 and Sr—O2 can be attributed to static or geometrical factors, specifically the rotation of the TiO_6_ octahedra. In contrast, the negligible difference in the MSRDs of Ti—O1 and Ti—O2 reflects the rigidity of the Ti—O bond. This observation further supports the conclusion that the TiO_6_ octahedra undergo rotation along the *c* axis.

### 3.5. Rotation angles of the TiO_6_ octahedra

**Figure 5:**
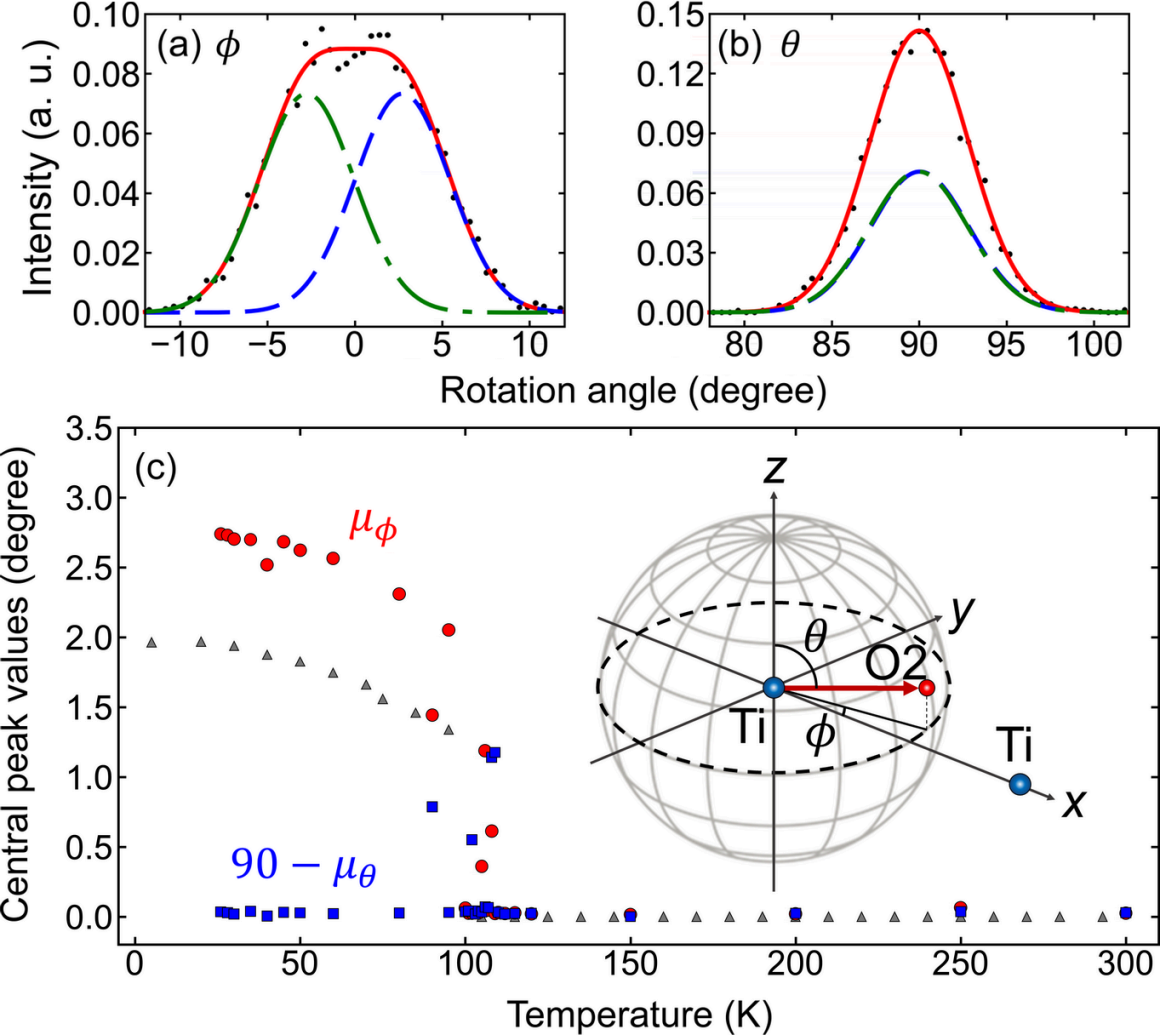
Probability density distributions of the (*a*) in-plane TiO_6_ rotation angle (ϕ) and (*b*) polar angle (θ) at 26 K (dots). A total of 3456 O2—Ti—Ti configurations are considered. The blue dashed and green dash-dot curves represent single Gaussian components; the red solid curves denote the bimodal Gaussian fitting results. (*c*) Temperature dependence of the central peak values: μ_ϕ_ (red circles) and 90 − μ_θ_ (blue squares). Neutron diffraction data from Hui *et al.* (2005 ▸) (gray triangles) are also presented. The inset in (*c*) illustrates the schematic of the relative polar coordinates (θ and ϕ).

The rotation angles of the TiO_6_ octahedron (θ, ϕ) were estimated using the polar coordinates of the Ti—O bond with respect to the Ti—Ti bond. Figs. 5 ▸(*a*) and 5 ▸(*b*) illustrate the probability density distributions of the in-plane rotation angle (ϕ) and the polar angle (θ) at 26 K, revealing a broader distribution for ϕ. The distributions at all temperatures were fitted with a bimodal Gaussian function,
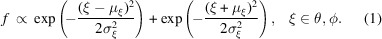
The central peak values (μ_ξ_) obtained from the fits are plotted as a function of temperature in Fig. 5 ▸(*c*). The fitting errors in μ_ϕ_ and μ_θ_ are 0.1° over the entire temperature range, except a larger error of 0.4° near *T*_C_ ± 5 K. Both θ and ϕ are zero above *T*_C_, indicating no rotation. Remarkably, only ϕ increases abruptly below *T*_C_, reaching 2.7° at the lowest temperature, while θ remains constant across the entire temperature range. The temperature dependence of ϕ aligns with predictions from Landau phenomenological theory (Hayward & Salje, 1999 ▸). Notably, the maximum rotation angle is 0.7° larger than the value of 2.0° reported by a previous neutron diffraction study (Hui *et al.*, 2005 ▸). This discrepancy arises from differences in the interatomic distances obtained from diffraction (*r*_D_) and EXAFS (*r*_E_) measurements. Specifically, the EXAFS technique, being locally sensitive, accounts for the perpendicular component of the MSRD (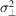) through the following expression (Sanson, 2021 ▸; Fornasini, 2001 ▸), 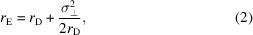
resulting in a slight relaxation of the local structure. Note that the results obtained by the RMC method, especially absolute values of parameters, depend on the initial conditions and constraints. As explained in the previous section, we carefully searched for the optimal *E*_0_ value and executed several RMC runs with different random number seeds to minimize the inevitable uncertainty.

### 3.6. Volumes of Voronoi polyhedra

**Figure 6:**
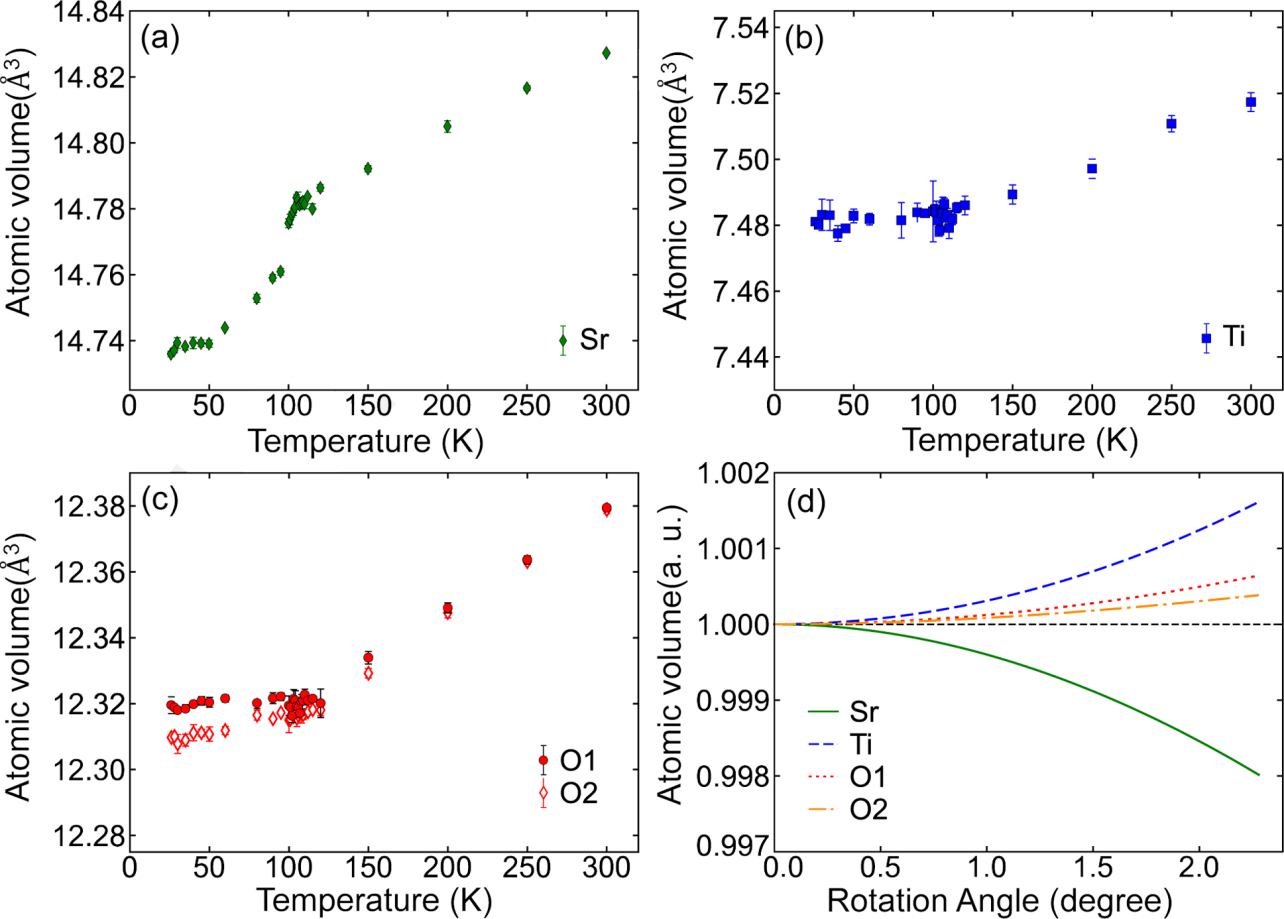
Temperature dependence of the Voronoi volumes of (*a*) Sr, (*b*) Ti and (*c*) O1 (red filled circles) and O2 (red open diamonds). (*d*) Voronoi volume of each atom in an ideal lattice plotted as a function of the TiO_6_ octahedral rotation angle.

Octahedral rotation can be analyzed from the perspective of Voronoi polyhedra, whose boundaries are defined by the perpendicular bisectors of neighboring atoms (Aurenhammer, 1991 ▸). The Voronoi volumes of each constituent atom, including inequivalent oxygen sites, are plotted in Fig. 6 ▸. The Voronoi volumes of all atoms increase steadily with temperature above *T*_C_. However, below *T*_C_, only the Voronoi volume of Sr decreases, mirroring the behavior of the MSRD for Sr—Sr, while those of Ti and O remain relatively constant. This stability in the Voronoi volumes of Ti and O provides clear evidence of the rigidity of the TiO_6_ octahedron. The softening of the Sr atomic vibration facilitates the rotation of the TiO_6_ octahedron (Yamanaka *et al.*, 2017 ▸; Tadano & Tsuneyuki, 2018 ▸). This conclusion is supported by a simplified model in which only the rotation of the TiO_6_ octahedron is considered, while other lattice parameters are held constant. The results, presented in Fig. 6 ▸(*d*), demonstrate a significant decrease in the Sr Voronoi volume.

### 3.7. Three-dimensional Gaussian fitting

**Figure 7:**
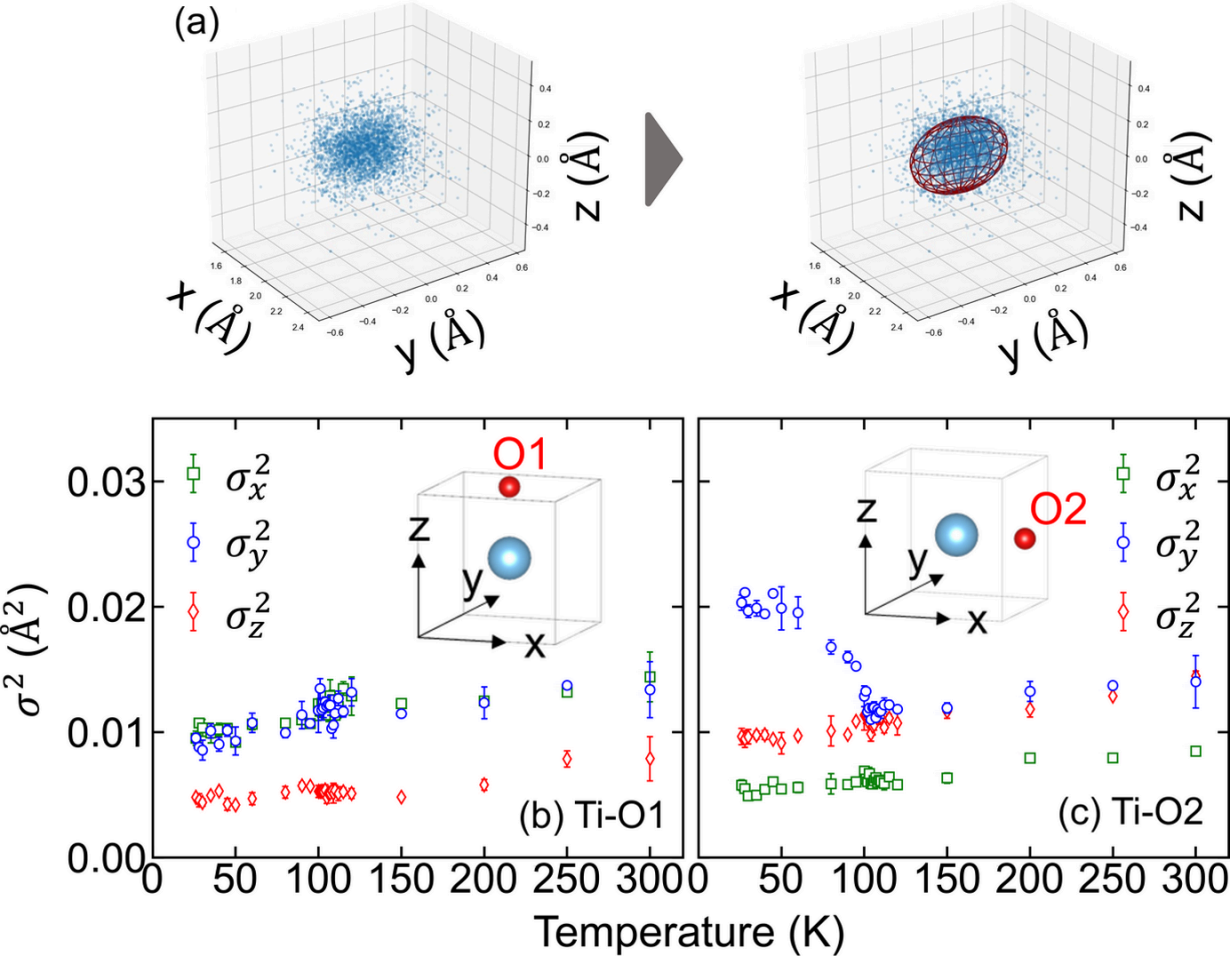
(*a*) Left: spatial distribution of O2 with respect to Ti at 26 K. Right: fitted ellipsoid based on a three-dimensional Gaussian function. (*b*) Square standard deviations [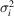 (*i* ∈ *x*, *y*, *z*)] for Ti—O1 along each direction as a function of temperature. Inset: coordinate definitions. (*c*) Squared standard deviations (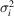) for Ti—O2 along each direction.

The spatial distributions of oxygen atoms relative to Ti in the clusters were modeled using the following three-dimensional Gaussian function,
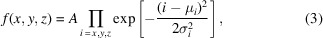
where *A*, μ_*i*_ and σ_*i*_ denote the scale factor, the mean positions and standard deviations, respectively. These were used as fitting parameters [Fig. 7 ▸(*a*)]. The temperature dependences of 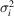 for Ti—O1 and Ti—O2 are plotted in Figs. 7 ▸(*b*) and 7 ▸(*c*), respectively. Note that the bond is directed along the *z* axis in Ti—O1 and along the *x* axis in Ti—O2, as illustrated in the corresponding insets. In both cases, 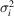 along the bond directions (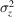 of Ti—O1 and 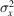 of Ti—O2) are the smallest among the three directions throughout the temperature range. This behavior reflects the rigid, covalent nature of the Ti—O bond (Cohen, 1992 ▸; Kuroiwa *et al.*, 2001 ▸). A key observation is that only the 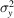 of Ti—O2 increases below *T*_C_.

Note that three-dimensional EXAFS analysis using two metal edges allows for the identification of the spatial distribution of light elements like oxygen, which is less feasible with conventional EXAFS methods. This three-dimensional analysis, analogous to a two-radar sensing method, is particularly effective for detecting angle distributions. The results of this study collectively confirm the rotation of the TiO_6_ octahedron and the volumetric reduction of the Sr Voronoi polyhedron.

## 4. Conclusions

In this study, we conducted a three-dimensional analysis of EXAFS spectra using the RMC method to investigate structural changes in each atom of STO during the cubic-to-tetragonal phase transition. The cluster generated from two-metal-edge fitting accurately reproduces the three-dimensional structure, including oxygen, which has been less extensively studied in EXAFS analyses. Our results revealed that the TiO_6_ octahedron rotates up to 2.7° below *T*_C_, driven by the loosely bound nature of the Sr cation. This work provides a framework for applying advanced structural analysis to other bimetallic oxides and nitrides, such as other *AB*O^3^-type perovskites as a large family of ferroelectric materials and ScGaN for a new type of lead-free ferroelectric material (Uehara *et al.*, 2024 ▸).

## Data Availability

Data supporting this publication are available upon reasonable request to the corresponding author.
